# Efficacy of long-term maintenance therapy with mycophenolate mofetil in lupus nephritis

**DOI:** 10.1186/2193-1801-3-638

**Published:** 2014-10-28

**Authors:** Zahra Rezaieyazdi, Tahmine Tavakoli, Mohammad Khajehdaluee, Shahram Honarmand

**Affiliations:** Rheumatic Diseases Research Center, Ghaem Hospital, School of Medicine, Mashhad University of Medical Sciences, Mashhad, Iran; Internal Medicine, Brigand University of Medical Sciences, Brigand, Iran; Community Medicine, Mashhad University of Medical Sciences, Mashhad, Iran

**Keywords:** Lupus nephritis, Mycophenolate mofetil, Glomerulonephritis

## Abstract

**Background:**

Mycophenolate mofetil (MMF) has long been used to manage lupus nephritis. Despite research on its long-term efficacy, it is still warranted to conduct further investigation regarding its indications, safety and outcome. This study was intended to evaluate our proposed protocol in maintenance therapy with MMF.

Twenty-four lupus nephritis patients were registered prior to their receiving 3–6 month induction therapy with monthly iv pulses of cyclophosphamide (CYC), followed by 24 month maintenance therapy using MMF and steroid. We defined end points as achievement of complete and partial remission, relapse, refractory to therapy as well as end stage renal disease (ESRD) and death. Friedman and repeated measurement tests were used to assess the effect of treatment on parameters over time.

Complete renal remission was achieved in 79.16% until the end of the last follow up with an average period of 12.45 ± 7.37 months since treatment commenced. Significant statistical differences were seen regarding proteinuria, hematuria, leukocyturia, plasma creatinine, C3, C4 before and after therapy (P < 0.05): plasma creatinine and proteinurea falling from 0.96 ± 0.65 to 0.75 ± 0.19 mg/dl (P < 0.14) and from 1.64 ± 1.12 to 0.27 ± 0.60 gr/24 h (P < 0.001). By the end of 24-month, 95.8% of patients had been in remission. Four episodes of relapse ended in remission followed by retreatment. No life-threatening side effects were observed in 66.6% of patients with fourteen cases of infection (58.3%). None of them developed ESRD.

Maintenance therapy with MMF was shown to yield favorable outcome with minimal complications, in treating lupus nephritis (IRCT2012071710313N1).

## Introduction

Renal involvement occurs in approximately 40% of patients with systemic lupus erythematosus. Nephritis is the first manifestation of lupus in 3–6% of patients and the severity of renal injury determines its prognosis. Generally, survival in lupus patients is roughly 92% at 10 years after diagnosis. Proliferative renal involvement is among the most severe manifestations of lupus and without proper treatment it can lead to significant morbidity and mortality (Cameron 1999; Mak et al. 2007; Korbet et al. 2007; Contreras et al. 2005; Bernatsky et al. 2006; Hahn et al. 2012).

A logical combination of drugs to achieve specific therapeutic goals is of paramount significance in treating lupus nephritis. The core of treatment is based on the application of drugs with maximum efficacy and minimum toxicity in order to control of nephritis to the point of allowing a good quality of life, and non progression of renal disease.

Effective therapy is designed to reduce mortality and prevent progress to end-stage renal disease. Immunosuppressive regimens of glucocorticoids combined with cytotoxic drugs, particularly cyclophosphamide, was effective and standard for the treatment of severe proliferative lupus nephritis (Hahn et al. 2012; Contreras et al. 2004; Ferrantelli et al. 2005; Flanc et al. 2004; Austin et al. 1986; Illei et al. 2001; Gourley et al. 1996; Steinberg and Steinberg 2005; Rezaie-Yazdi et al. 2005; Mok et al. 2001; Ioannidis et al. 2000; Grootscholten et al. 2007). However, cyclophosphamide has both instant and cumulative adverse effects, including marrow suppression, gonadal toxicity, hemorrhagic cystitis, and the increased risk of cancer as well as the possibility of no response or relapse in several patients (Houssiau et al. 2002, 2010a). Therefore, other therapeutic agents, such as azathioprine and mycophenolate mofetile with few toxic effects, come prior to other alternatives (Grootscholten et al. 2006; Arends et al. 2012; Sahin et al. 2008; Houssiau et al. 2010b; Dooley et al. 2011).

Nowadays mycophenolate mofetile has been considered an important alternate agent for refractory lupus nephritis with hopeful results and reasonable side effects (Chan et al. 2000, 2005; Lenz et al. 2005; Weng et al. 2010; Zhu et al. 2007; Ong et al. 2005; Karim et al. 2005). Mycophenolic acid, the active metabolite of mycophenolate mofetil , selectively suppresses the proliferation of T and B lymphocyte, the formation of antibodies, and the glycosylation of adhesion molecules by inhibiting purine nucleotide synthesis and depleting lymphocytes and monocytes of guanosine triphosphate (Eickenberg et al. 2012). Mycophenolate mofetile as maintenance therapy after short-term intravenous cyclophosphamide have been shown efficient and safe, reducing the long-term exposure to cyclophosphamide (Bernatsky et al. 2006; Flores-Suárez 2006; Borba et al. 2006; Tse et al. 2006). In comparison with cyclophosphamide the adverse effects of MMF have been revealed to be well- tolerated, with gastrointestinal upset being the most common, and no mutagenic effects (Laskari et al. 2010; Elyan and Ballou 2009).

The aim of our study was to evaluate the efficacy and safety of mycophenolate mofetil combined with prednisolone for maintenance treatment of lupus nephritis in a single center cohort of patients with proliferative lupus nephritis.

## Patients & methods

In this open label clinical trial twenty four consecutive patients with the diagnosis of lupus in accordance with ACR classification criteria (Tan et al. 1982) were enrolled and prospectively followed up during 24 months. All patients had the criteria for nephritis. Renal biopsy was done for 20 patients. Every specimen was observed under light microscopy, the findings of which were subsequently categorized based on the revised World Health Organization (WHO) classification for lupus nephritis (Weening et al. 2004).

Inclusion criteria were defined by one of the followings:

- Evidence of active proliferative glomerulonephritis in the renal biopsy (WHO class IV, III)
- In case of absent renal biopsy or the presence of WHO class V in biopsy, with the presence of the following clinical or paraclinical findings:
  - Proteinaria >1 gr/24 h
  - Progressive renal failure with 30% decrease in creatinine clearance over one-year period and creatinine >1.9 mg/dL
  - The presence of more than 5 red cells in HPF of urine sediment in two separate specimens taken in a year, presence of WBC, granular or hyaline casts without active infection (was examined under conventional light microscope)

Exclusion criteria include:

WHO class I or II lupus nephritis, end stage renal disease when replacement renal therapy will be indicated, leukopenia (neutrophils < 1500/mm3) due to bone marrow suppression, recurrent episodes of bacterial infection, history of cytotoxic drug treatment for more than two weeks or pulse therapy with corticosteroids during a six-week period before study entry.

### Study protocol and immunosuppressive treatment

For classes III and IV of lupus nephritis, the treatment considered as four phases:

1. Induction: intravenous cyclophosphamide, given as boluses once a month for 3–6 consecutive months (to the maximum dose of 1 g/m2) in addition to corticosteroid. All patients received ondansetron to prevent nausea and vomiting with every CYC pulse. High dose glucocorticoid was initiated with ivtravenous pulse methylprednisolon (500–1000 mg given over 30 minutes daily for three days) then changed to oral prednisolon (40–60 mg) for one month, tapered to intermediate dose during maintenance phase of therapy.
2. Maintenance: mycophenolate mofetil (cellcept®, manufactured by Zahravi Pharm.Co, Under License of F. Hoffmann-La Roche Ltd, Basel, Switzerland) to the maximum dose of 2 gr/day combined with corticosteroid.
3. Tapering: mycophenolate mofetil dosage remains unchanged for the first year followed by dose reduction in the second year, in the absence of relapse or partial remission fulfillment.
  When partial remission was achieved, we started to taper prednisolon by an average of 10% monthly in order to reach an optimum dose of 5–10 mg/day.
4. Discontinuation: if our study goals had been achieved, the mycophenolate mofetil would have been discontinued with the further tapering of prednisolone to the lowest possible daily dose and patients’ follow-up for evidence of relapse.

Those with class V lupus nephritis were given mycophenolate mofetil combined with corticosteroid since the diagnosis was made.

Patients were followed up every month during induction therapy and every other month during the first year and every three months thereafter. During each visit, the patients were evaluated including a complete physical examination as well as all laboratory and serologic tests (blood count, urine analysis, creatinine, GFR, measurement of proteinuria in 24 h urine collection, C3, C4, Anti-DNA, ANA, ESR). At each visit, side effects were investigated via enquiry and thorough physical examination. Each patient had a complete clinical evaluation for any other organ involvement besides nephritis as well as episodes of relapse.

Informed consent was obtained from all patients. The design of the work has been approved by the ethical committee of Mashhad University of Medical sciences.

### End points were defined as

Criteria for complete remission:

- Complete improvement of renal and extra-renal symptoms:
  Rise of creatinine less than 0.3 mg/dL, less than 300 mg proteinuria per day (only trace proteinuria) or less than 50% of initial proteinuria when Cr >1.7 mg/dl, absence of RBC cast, complete regression of all systemic symptoms
- Return to within normal limits of ESR, C3, C4, Hb and fall of autoantibody titers
- Absence of relapses and infectious complications

Criteria for partial remission:

- No progression of renal disease (normal or stable renal function)
- Decrease of at least 50% in dysmorphic RBC, cellular casts and proteinuria in the absence of doubling of serum creatinine or less than 1 gr proteinuria per day
- Return to within normal limits of markers of inflammation such as ESR
- Regression of systemic symptoms
- Return of patient to functional class 2 in the presence of an acceptable rate of complications

Relapse:

- More than 50% increase in (after reaching the lowest level during therapy) serum creatinine, dysmorphic RBC, cellular cast
- Doubling of proteinuria if there was nephritic proteinuria or at least 2 gr/day if based proteinuria < 3.5 gr/day
- At least two systemic symptoms reappeared

Refractory to therapy:

- No renal response in spite of 6 month treatment with mycophenolate mofetil

End stage renal disease:

- Plasma creatinine rose and got stabilized above 5 mg/dl for 3 months

### Statistical analysis

Analyses of all data were conducted in SPSS version 11.5. The significance of the process of changes of variables with abnormal distribution was assessed by Friedman test whereas those with normal distribution were tested by repeated measurement.

Wilcoxon signed ranks test was used for comparing quantitative variables with abnormal distribution while Paired t-test was applied to variables with normal distribution. All mean values were shown ±1SD and P values < 0.05 were considered statistically significant.

## Results

### Patients’ characteristics

We enrolled 20 females and 4 males with active lupus nephritis with majority documented with diffuse proliferative glomerulonephritis. Two tissue samples (10%) were compatible with WHO class III, 13 samples were WHO class IV (65%) and 5 samples were WHO class V (25%). Adverse predictive factors such as proteinuria, low GFR, hypertention, were detected in 95.8%, 33.3% and 41.6% of patients respectively. At the beginning of renal disease 21 patients had hematuria and 18 patients had leukocyturia. The average range of proteinuria before treatment was 1.57 ± 1 gr/day. Three patients (13%) had nephrotic syndrome (proteinuria >3gr/day). Decrease in C3 and C4 levels were seen in 33.30% at the beginning of therapy. Anti DNA was positive in 50% of patients. Renal insufficiency means creatinine >1.9 mg/dL was seen in one patient at the beginning of therapy.

Extra renal manifestations at baseline are as follows: skin rash 13(54.2%), arthritis 15(62.5%), oral ulcer 16(66.7%), vasculitis 3(12.5%), nervous system involvement 3(13%), cardiac involvement 1(4.2%) and pneumonitis 1(4.2%). Baseline characteristics of 24 patients with lupus nephritis are shown in Table 1.

**Table 1:** **Characteristics of twenty four patients with lupus nephritis**

Characteristics	Mean	Range
Age (year)	20.7 ± 8.1	10 – 40
Duration between onset of lupus and renal involvement (month)	15.17 ± 22.79	0 – 84
Serum Creatinine (mg/dl)	0.95 ± 0.63	0.10 – 3.5
GFR(ml/min/1.73 m^2^)	83.19 ± 32.99	20.40 – 162.91
Urinary protein (g/24 h)	1.57 ± 1.11	0.10 – 4.3
Serum C3 (mg/dl)	63.12 ± 28.46	14 – 120
Serum C4 (mg/dl)	17.19 ± 8.38	6-35
Platlete (number/mm^3^)	255 ± 93 × 10^3^	63 – 511× 10^3^
Serum Hb (mg/dl)	12.01 ± 1.72	8.8 – 15.9
ESR/h	32.79 ± 28.08	4 – 130
Systolic BP (mmHg)	129.58 ± 22.69	80 – 180
Diastolic BP (mmHg)	86.04 ± 14.25	60 – 120
Urine WBC (n/hpf)	19.25 ± 27.38	0 – 100
Urine RBC (n/hpf)	21.87 ± 31.15	0 – 100

**Figure 1:**
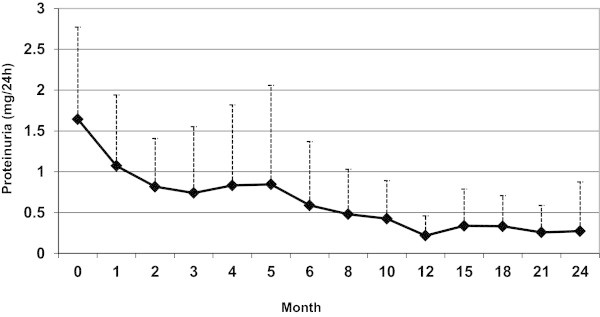
**Proteinuria and its changes during the course of follow up.**

**Figure 2:**
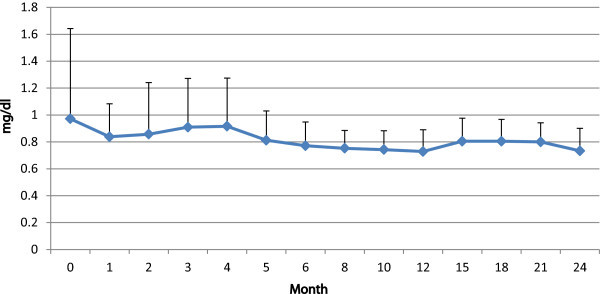
**Creatinin and its changes during the course of follow up.**

**Figure 3:**
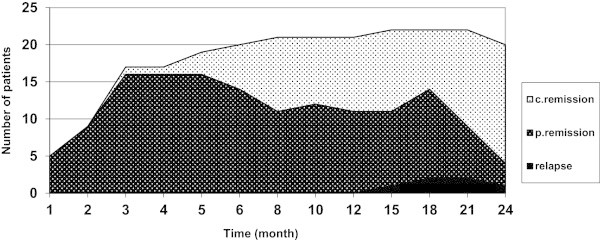
**The process of changes of patients responses to treatment during the course of follow up.**

Results of the statistical comparisons among selected variables at baseline and at the latest follow up showed a significant improvement of all parameters including proteinuria, C3 and C4 (Table 2). Then we evaluated laboratory data variables changes during the course of treatment.Significant alterations could be observed in proteinuria which resolved in 20 out of 24 (83.3%) patients (Figure 1), and hematuria (P < 0.001), whereas parameters such as platlete count, Hb and ESR seemed immune to drastic changes (P = 0.31, P = 0.88, P = 0.24). The creatinine level changes during the course of therapy has been shown in Figure 2 (P = 0.68). As to renal involvement implications systolic and diastolic blood pressure varied in their pattern of, with the latter falling substantially (P = 0.02) while the former remained almost unchanged (P = 0.09).As can be seen in the Figure 3, initial response to therapy began since the first month following drug administration in accordance with our protocol. Sixteen cases were shown to match partial remission criteria at the end of the third month, and one patient achieved full remission; which could first be seen at the end of the third month, gaining momentum at a sharp pace to peak at around month 21.

**Table 2:** **Statistical comparisons of selected variables prior to and following treatment**

Parameters	Before (mean ± SD)	After (mean ± SD)	P value
Proteinuria(gr/24 h)	1.57 ± 1.11	0.27 ± 0.60	<0.001
Hematuria(n/hpf)	21.87 ± 31.15	2.28 ± 3.42	<0.001
Leukocyturia(n/hpf)	19.25 ± 27.38	2.85 ± 5.59	0.001
C3(mg/dl)	63.12 ± 28.46	97.79 ± 20.73	<0.001
C4(mg/dl)	17.19 ± 8.38	23.41 ± 8.47	0.006
Creatinine(mg/dl)	0.96 ± 0.95	0.75 ± 0.19	0.144
GFR(ml/min/1.73 m^2^)	83.19 ± 32.99	98.96 ± 26.73	0.060

### Outcome measures

#### Partial renal remission

With a mean renal remission time of 3.67 ± 2.58 months from baseline, five out of 24 patients (20.83%) reached partial renal remission.

#### Complete renal remission

Complete renal remission was achieved in 19 out of 24 patients (79.16%) at the end of the last follow up. Average period of complete remission was 12.45 ± 7.37 month since the beginning of treatment.

At the end of follow-up, 23 out of 24 patients (95.8%) of the patients had reached remission.

#### Renal relapse

Relapses occurred during the maintenance phase of therapy in 4 out of 24 patients in remission (16.6%).

Relapses happened at 15^th^, 18^th^ and 21^st^ months of therapy and all patients except one responded when retreated with cyclophosphamide pulse followed by mycophenolate mofetil.

#### Chronic renal failure-death

There was no refractory-to-therapy case. None of our patients developed chronic renal insufficiency or died.

#### Extra renal manifestation and pregnancy

The majority of the initial extra renal manifestations resolved during the maintenance phase of therapy. None of our patients developed sustained amenorrhea. During this treatment course, we had four patients who had been pregnant at 8^th^, 10^th^, 15^th^ and 21^th^ months since treatment commenced, when they were in partial remission, ending in unproblematic delievery. Azathioprine replased mycophenolate mofetil throught pregnancy period, with one relapse episode following delivery.

#### Side effects

Side effects were observed in sixteen out of the 24 patients (66.6%) with fourteen cases of infection (58.3%).

No hemorrhagic cystitis was observed while transient gastrointestinal complications affected two patients during maintenance therapy (12%) (diarrhea, gastrointestinal discomfort and nausea).

## Discussion

Mainstream therapy is primarily intended to improve renal function as well as to prevent progressive disease. Mycophenolate mofetil was approved for clinical use in lupus nephritis over the past years. Several studies have been conducted on mycophenolate mofetil for maintenance therapy of lupus nephritis (Hahn et al. 2012; Sahin et al. 2008; Houssiau et al. 2010b; Dooley et al. 2011; Chan et al. 2000, 2005; Lenz et al. 2005; Weng et al. 2010; Karim et al. 2005; Flores-Suárez 2006; Borba et al. 2006; Tse et al. 2006; Laskari et al. 2010; Elyan and Ballou 2009; Ginzler et al. 2005; Mak et al. 2009).

On the basis of anecdotal reports of success with mycophenolate mofetil in patients with lupus nephritis with a considerable likelihood for poor outcomes (Chan et al. 2000, 2005; Lenz et al. 2005; Ginzler et al. 2005), in the present study we intended to examine the safety and efficacy of MMF as maintenance therapy for proliferative lupus nephritis following a short-term induction therapy with iv CYC. Favorable response with acceptable side effects were observed in most patients.

Of the 24 patients treated based on our therapeutic protocol, partial as well as full remission could be achieved in a significant percentage, 95.8% of the patients, while relapse rates were as low as 16.6%. Our study was free of severe complications such as renal failure and mortality. We had the acceptable rate of relapse, 16.6% after the 15^th^ month, during the course of therapy, all of whom treated with short-course cyclophosphamide pulse therapy and mycophenolate mofetil to achieved remission. All cases of relapse were seen one year following the last pulse of cyclophosphamide in the third phase of therapy when MMF dose was reduced. This may indicate that our patients might have needed a longer period of treatment in the second phase. The dose of prednisolon has also been reduced in the third phase. Relapses could have been delayed had steroid been tapered at a slower pace.

One interesting point regarded successful pregnancies among our patients thought our course of therapy. These cases were in 10^th^, 15^th^and 21^st^ month of treatment when the patients were in partial remission. Another case was in her 8^th^ month and in complete remission but lupus nephritis relapsed following delivery.

The response rate to MMF therapy in this study was significant and compatible with other reports. Similar to Chan et al. study on 21 and 33 patients who showed 81% and 72.7% complete remission with MMF therapy (Chan et al. 2000, 2005).

In the study of Elyan and Ballou (Elyan and Ballou 2009) on 25 patients with lupus nephritis treated with MMF, 57% and 17% of patients achieved complete remission and partial remission in order. On average it took our cases 3.6 and 12.4 months to achieve partial and full remission respectively whereas Elyan and Ballou reported an average of 8.5 month. Results of the statistical comparisons among hematuria, leukocyturia, proteinuria, C3 and C4 showed significant differences before and after treatment, with GFR and creatinine changing slightly which was similar to Elyan and Ballou study (Elyan and Ballou 2009).

As our only three cases of relapse occurred throughout the tapering phase, we may safely conclude that early reduction in steroid and mycophenolate mofetil dosage can account for them.

However, one case of relapse following delivery can also be explained owing to replacing MMF with azathioprine.

Alteration trends pertaining to laboratory finding variables during the course of treatment were also shown almost parallel with other reports findings (Laskari et al. 2010; Elyan and Ballou 2009). Ginzler EM et al. published multicenter, randomized clinical trial and showed that MMF is an appropriate alternative to CYC for the treatment of renal diseases in patients with biopsy-proven lupus nephritis (Ginzler et al. 2005). Our study was in agreement with their observations.

In Laskari et al. (2010) study from Greece, thirty-three consecutive patients with proliferative lupus nephritis received oral MMF 2 g/day as maintenance therapy for a median time of 29 months. They showed a significant improvement of all renal parameters at the end of the induction treatment as well as at the latest follow-up compared to the baseline. Renal remission achievement rate to the end of the follow-up was 73% whereas it was 54% for complete remission cases. They reported 4 (12%) patients who relapsed within 19–39 months after initial response. At the end of their follow-up, 51% of patients had reached remission. Their results were in agreement with ours except for the fact that we had fewer side effects in comparison with the Laskari et al. findings (Laskari et al. 2010).

In our clinical trial, there were only two gastrointestinal complications due to MMF which is generally reversible and in comparison with cyclophosphamide side effects, seems insubstantial. The majority of women preserved ovarian function, with four pregnancies. Bone marrow suppression was not a complication of MMF in our study. In contrast to the study by Contreras et al., in which 1 episode of chronic renal failure and 1 death due to severe infection happened, such outcomes were not observed in our cases (Contreras et al. 2004). Some advantages of this study with regard to well defined criteria for examined parameters include lower dose of MMF and long-term follow-up with regular intervals.

The present study suggests that small dose MMF combined with the shortest duration of CYC therapy may be safer than long-term use of CYC without compromising efficacy. This investigation had not been intended for evaluating the effectiveness of MMF as induction therapy in proliferative lupus nephritis. However, there have been reports highlighting the role of MMF in inducing remission (Zhu et al. 2007; Mak et al. 2009; Lee et al. 2010; Walsh et al. 2007; Hui et al. 2013). According to previous data a better outcome with MMF has been demonstrated for non-Caucasian patients (Lee et al. 2010), with considering the fact that our patients were Caucasian, the present study results may put emphasis on the benefit and safety of long term use of MMF in Caucasian race. Future large cohort study of lupus nephritis patients with well defined and strict criteria for all examined parameters such as criteria for full remission, partial remission, relapse, flare as well as the opportunity to have long period of regular follow up would establish our observations.

We were also restricted in terms of the following: As it was an open label clinical trial, there was no control group then the possibility of randomization and masking was not considerable. As continuous follow up and repeated observations of variables in different time interval was performed, so there was possibility of measurements biases due to mean reversion phenomenon. Moreover, the limited numbers of patients made it impossible to generalize our findings in Caucasian race. Other factors that limited our study were the lack of any plan for re-biopsy after course of treatment.

On the other hand, our study contain valuable data on the main concern of maintenance treatment in lupus nephritis given the long period of follow up, the clear design and regular follow up of all patients. Randomized controlled trials containing a larger group could corroborate our findings.

In sum, we concluded that mycophenolate mofetil appear to be efficacious and very safe as maintenance treatment for proliferative lupus nephritis following an intensive induction therapy with a short-course of monthly iv, endoxan pulse. That improved renal remission and reduce relapse rates as well as reduction in cyclophosphamide toxicity.
